# Low- and high-cocaine intake affects the spatial and temporal dynamics of class IIa HDAC expression-activity in the nucleus accumbens and hippocampus of male rats as measured by [18F]TFAHA PET/CT neuroimaging

**DOI:** 10.1016/j.addicn.2022.100046

**Published:** 2022-11-08

**Authors:** Shane A. Perrine, Walid F. Alsharif, Arman Harutyunyan, Swatabdi Kamal, Nerissa T. Viola, Juri G. Gelovani

**Affiliations:** a Psychiatry and Behavioral Neurosciences, Wayne State University, 6135 Woodward Avenue, Suite 3119, Detroit, MI, USA; b Research Services, John D. Dingell VAMC, Detroit, MI, USA; c Biomedical Engineering, Wayne State University, Detroit, MI, USA; d Oncology, Wayne State University, Detroit, MI, USA; e Karmanos Cancer Institute, Detroit, MI, USA

**Keywords:** Cocaine, Rat, Epigenetics, Histone deacetylase, Positron emission tomography

## Abstract

Repeated cocaine alters neuronal function in the nucleus accumbens (NAc), a brain region involved in cocaine taking, and in hippocampus (HC), known for contextual and associative learning. [^18^F]TFAHA is a histone deacetylase (HDAC) class IIa-specific radiotracer for positron emission tomography (PET)-imaging developed by our group to study epigenetic mechanisms. Here, [^18^F]TFAHA was used to conduct PET-imaging coupled with computed tomography (CT) of rat brains at baseline and after repeated cocaine intravenous self-administration (cocaine-IVSA) in low-intake versus high-intake cocaine groups. A 3 h-access FR1-schedule of cocaine-IVSA (0.5 mg/kg/infusion) for 12 continuous days was used with male Sprague Dawley rats following jugular vein catheterization. PET/CT neuroimaging with [^18^F]TFAHA was acquired in a dynamic mode over 40 min post-radiotracer administration at baseline and on day 12 of cocaine-IVSA using a longitudinal, repeated design. This study shows that high-cocaine intake significantly decreases class IIa HDAC expression-activity in NAc, while low-cocaine intake significantly decreases expression-activity in HC in male rats. These findings suggest the individual rats with low-cocaine intake had epigenetic changes in HC, where drug-associative changes occur. Alternatively, individuals with high-cocaine intake had robust epigenetic changes in NAc, where rewared-related behaviors originate. These findings are the first longitudinal data obtained *in vivo* to implicate class IIa HDACs in the persistent behavioral effects of cocaine. Furthermore, our results are consistent with published research implicating class IIa HDACs in cocaine-induced brain changes and studies suggesting a relationship between an individual’s drug-taking behavior and regional pattern of epigenetic changes in the brain.

## Introduction

Abuse and dependence on cocaine remain a significant mental healthcare problem with no treatments for cocaine use disorder despite decades of unsuccessful drug testing. This emphasizes the need to understand neuronal mechanisms that mediate neuroplasticity caused by repeated cocaine to facilitate evidence-based neurotherapeutic development. Research in animal models has implicated epigenetic mechanisms in the persistent effects of cocaine and other drugs of abuse on addiction-related behaviors [1]. The class IIa histone deacetylases (HDACs), including HDAC4, HDAC5, HDAC7 and HDAC9, uniquely regulate chromatin modification and epigenetic reprogramming [2-5]. Research shows class IIA HDACs, notably HDAC4 and HDAC5 in the nucleus accumbens (NAc), play a key, yet complex role in the reinforcing and rewarding effects of cocaine by regulating transcription of factors involved in promoting neuroplasticity [5-14].

Cocaine intravenous self-administration (cocaine-IVSA) is the gold-standard rodent model of addiction-related behaviors [2,15]. The NAc drives motivated cocaine-taking behaviors, and the hippocampus (HC) controls the contextual association during operant behavior within the self-administration chamber [16]. In addition, both motivated behavior and contextual association are regulated by epigenetic-based transcriptional changes, including modulation by class IIa HDACs, in the NAc and HC [17-19].

As previously indicated, cocaine affects class IIa HDAC activity, mostly by downregulating levels of HDAC4 and HDAC5 in the brain regions of the reward system [5-14]. However, these studies have relied on conventional immunoassays to determine the level of expression or used viral vector approaches to study subcellular localization of these enzymes. The effects of cocaine on the enzymatic activity of class II has heretofore not been studied. Therefore, the goal of this study was to assess the spatial and temporal dynamics of class IIa HDAC expression and activity in the NAc and HC *in vivo* and non-invasively using advanced molecular imaging methodology in rats undergoing chronic cocaine-IVSA.

[^18^F]TFAHA is a substrate-based radiotracer used to assess the expression-activity of class IIa HDAC epigenetic enzymes *in vivo* [20]. [^18^F]TFAHA enters cells where it can be selectively-cleaved by class IIa HDACs, despite their relatively weak deacetylase activity [21], leaving the cleaved radiolabeled ^18^F-trifluoroacetate group metabolically trapped inside the cell. Previous positron emission tomography (PET)-imaging studies in naïve rats demonstrated highly selective accumulation of [^18^F]TFAHA in the NAc and HC, consistent with high levels of HDAC4 and HDAC5 expression and activity in these brain regions [20,22]. Furthermore, using PET/CT-imaging with ^18^F-TFAHA a significant decrease in HDACs class IIa expression-activity was observed in the brain of rats after mild traumatic brain injury (mTBI) [23]. Here, we hypothesized that longitudinal PET/CT neuroimaging will be sensitive enough to demonstrate that cocaine intake decreases the accumulation of [^18^F]TFAHA in the NAc based on previous studies. If successful, such neuroimaging methodology can readily be applied in clinical studies in human subjects.

## Methods

### General methods

**Fig. 1.**
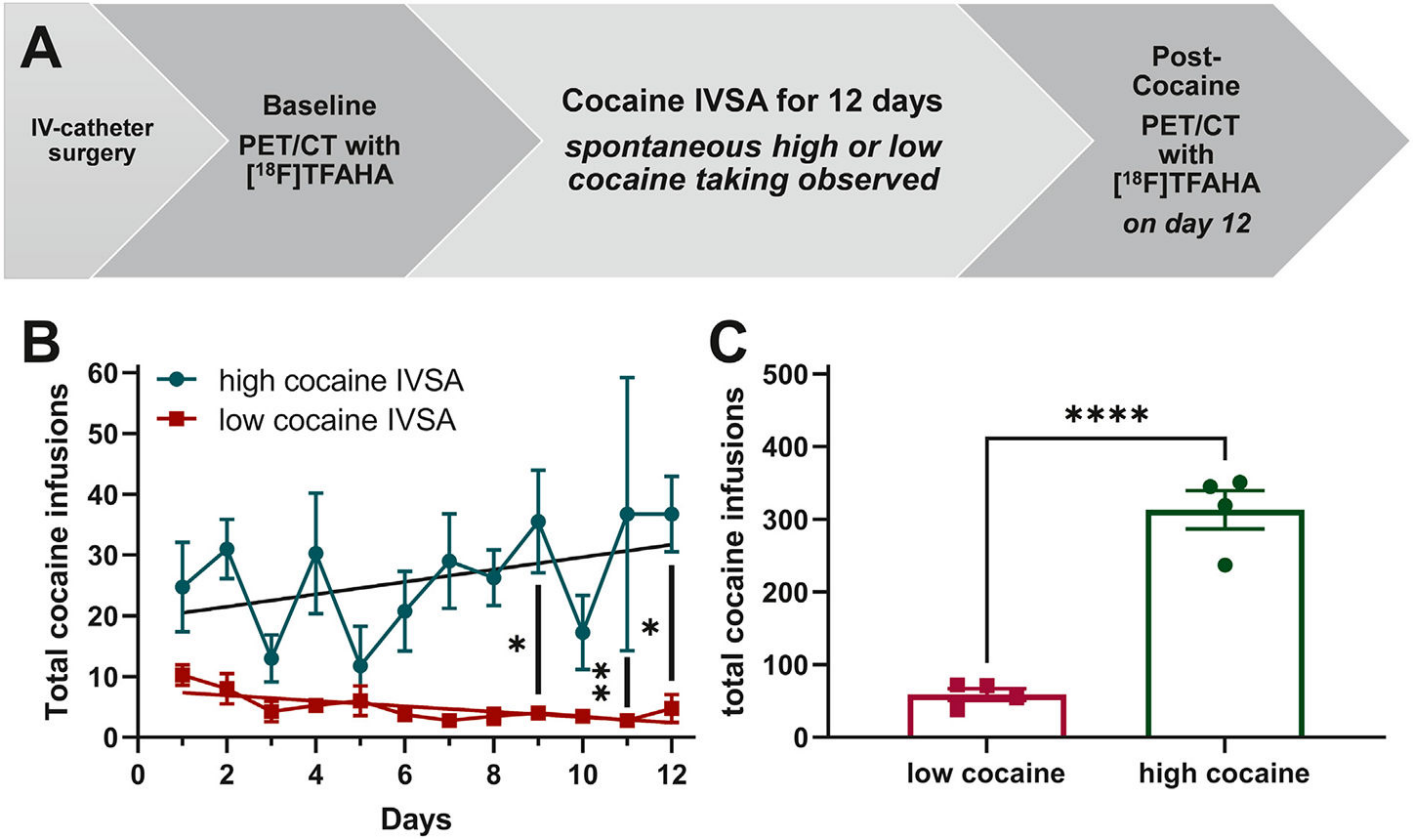
Intravenous self-administration (IVSA) of cocaine by rats taking high cocaine or low cocaine infusions. (A) A schematic of the experimental timeline is shown. Male Sprague-Dawley rats underwent jugular vein catheterization and recovery, followed by a baseline PET/CT with [^18^F]TFAHA, then they were allowed to self-administer cocaine (0.5 mg/kg/infusion) in 3 h daily access sessions under fixed-ratio 1 (FR1) conditions for 12 consecutive days, and finally, re-scanned post-cocaine on day 12 using PET/CT with [^18^F]TFAHA. (**B)** The total number of cocaine infusions (daily mean ± s.e.m. with linear regression line) over the 12 days is significantly different between subsets of rats. The data show that a subset of rats exhibit high cocaine taking while other rats display low cocaine taking behavior. (**C)** The total number of cocaine infusions plotted as the sum of daily activity (mean ± s.e.m. or individual data points) over the 12 days shows that a group of rats (i.e., high cocaine) spontaneously take significantly more cocaine than another group of rats (i.e., low cocaine). The high cocaine taking group (n=4 rats) is shown in green and circle symbols, while the low cocaine taking group (n=4 rats) is shown in red and square symbols; *p<0.05, **p<0.01, ****p<0.0001.

All experiments were conducted at Wayne State University (WSU) in Detroit, MI. Before initiating studies, the procedures were approved by the WSU Radiation Safety program, which holds a Type A broad-scope license with the Nuclear Regulatory Commission, and the WSU Institutional Animal Care and Use Committee, which adheres to principles, policies, and procedures outlined in the *Guide for the Care and Use of Laboratory Animals* [24]. Fig. 1A illustrates the experimental timeline.

Male Sprague–Dawley rats (Charles River Laboratories, Portage, MI; >250 g or older than postnatal day 55) were allowed to acclimate in pair-housing to the vivarium for at least 5 days following arrival before experimentation. Following surgical catheterization, rats were single-housed for the remainder of the study in standard microisolator rat polycarbonate cages with bedding throughout the experiment. Animals were allowed food and water ad libitum in their home cages and housed on a 12 h light/dark cycle with lights on at 6AM. Temperature and humidity were controlled in the vivarium and behavioral testing room. The study used two cohorts of rats (4 rats/cohort) to stagger PET-imaging sessions.

(−)Cocaine hydrochloride was provided free by the National Institute on Drug Abuse (NIDA) Drug Supply Program (Bethesda, MD) and supplied by Research Triangle Institute (Research Triangle Park, NC) to SAP, who holds U.S. Drug Enforcement Administration and State of Michigan schedule II-V controlled substance licenses to conduct research in rodents. Cocaine was dissolved in sterile saline (0.9% NaCl) and delivered intravenously (IV) during the cocaine self-administration procedure (i.e. cocaine-IVSA).

### Cocaine-IVSA

Rats were surgically catheterized via the jugular vein using aseptic conditions with a single chronic indwelling catheter under ketamine (90 mg/kg intraperitoneal, IP) and xylazine (10 mg/kg IP) anesthesia. Carprofen (5 mg/kg subcutaneous, SC) was administered immediately before and daily for 1–2 days post-surgery, as needed. Catheters were threaded SC under the skin and attached to stainless steel tubing in a mesh tether button, exiting through a 1 cm incision between the scapulae. Catheters were flushed daily with 0.5 ml of heparinized saline (50 U/ml) to maintain catheter patency and gentamicin as needed. Rats were given at least 5 days to recover from surgery before conducting baseline PET-imaging session.

All self-administration sessions were conducted in operant conditioning chambers located in sound-attenuating chambers using commercially available supplies (Med Associates Inc., St. Albans, VT). Chambers were equipped with two nose-poke (NP) devices positioned 3 cm above the stainless steel grid floor on one wall and a white house light at the top of the opposite wall. A variable rate infusion pump delivered cocaine infusions through tubing held by a spring tether connected to a swivel-joint on a counter-balanced arm. Rats were tethered in the operant chambers during each cocaine-IVSA session. When not participating in cocaine-IVSA, rats were housed in the vivarium.

Acquisition of cocaine-IVSA sessions began with illumination of a yellow light in the active (cocaine) NP response only. Responses in the active NP were recorded and produced a single cocaine infusion of 0.5 mg/kg/infusion (100 ml/kg/s infusion) under a fixed ratio 1 (FR1) schedule of reinforcement. A response in the active NP coincided with the illumination of the house light and extinguished the NP light for a 20 s time-out during which no cues were available, but NPs were recorded. Rats continued on the FR1 schedule daily for 12 consecutive sessions, each lasting for 3 h. Responses in the inactive NP were recorded but had no programmed consequence. The daily and total (summed) infusions of cocaine were measured. The daily and total (summed) active or inactive NP responding during drug-availability and active NP responding during timeouts were collected. Correct responding was determined as active NP divided by the sum of active and inactive NP, averaged across all cocaine-IVSA days, and expressed as a percentage.

### PET-imaging using [^18^F]TFAHA

The radiosynthesis and formulation of [^18^F]TFAHA were performed as previously described [20,22]. [^18^F]TFAHA-PET/CT-imaging was performed at baseline and on day 12 within 2 h of the cocaine-IVSA session on that day. Rats received 2-4% isoflurane (in O_2_ at 0.6 L/min) throughout the imaging procedure while maintaining body temperature at 37°C with an electronically-controlled heating pad (M2M Imaging, Cleveland, OH). Rats were secured in the supine position on the bed of a microPET R4 scanner (Siemens, Knoxville, TN) with the brain in the center of the field of view. A total of 300–500 *μ*Ci of [^18^F]TFAHA in 1 ml sterile saline was administered per animal via the tail-vein intravenous injection over 1 min and dynamic PET-images were acquired for 40 min. After PET-imaging, the bed with the secured, anesthetized animal was transferred to an Inveon SPECT/CT scanner (Siemens, Knoxville, TN). Whole-body CT images were acquired with 4 overlapping frames (2 min each) with X-ray tube settings of 80 kV and 500 μA. PET-images were reconstructed using an ordered subset expectation–maximization method [20,22]. The digital-version of the Paxinos and Watson Rat Brain Atlas was used for image alignment and identification of brain regions of interest (ROIs), specifically NAc and HC, and neuroanatomical markers [25]. The levels of class IIa HDAC dependent [^18^F]TFAHA accumulation in different ROIs were analyzed using Amide’s Medical Imaging Data Examiner tool (AMIDE v1.0.4 Software) and expressed as standard uptake values (SUV) [20,22]. Logan graphical analysis for reversibly binding (or accumulating) radiotracers was used to calculate the distribution volume (DV) of [^18^F]TFAHA-derived radioactivity in the NAc and HC using the cortex as a reference tissue [20,22]. Following PET/CT-imaging at baseline, rats were quarantined for at least 3 days to allow for radioactive decay before starting cocaine-IVSA. Rats were euthanized following the second, post-cocaine PET/CT-imaging session.

### Statistical analyses

All data were analyzed using Excel 2016 (Microsoft Corporation) and statistical analyses were conducted and graphs prepared using Prism 9 (GraphPad Software LLC). Two-way repeated-measures analysis of variance (2-way RM-ANOVA) using cocaine group (low-cocaine or high-cocaine) and time (all 12 days) as main factors with matching values stacked, and full interaction modeling was used to analyze the total infusions of cocaine over time with Bonferroni’s multiple comparisons post hoc test. Simple linear regression lines were determined for these data and plotted in Fig. 1B for the high cocaine taking group. Additionally, the total cocaine infusions, total active or inactive NP responding for all 12 days of behavior (i.e., total responding), the average percent correct responding, and the average responding on the cocaine NP during time-out data were compared, individually, between low-cocaine and high-cocaine groups using two-tailed, unpaired *t*-test. Data from Logan graphical analyses were plotted and fitted using a non-linear, least-squares regression with no weighting or constraints on data. A 2-way ANOVA was used to analyze [^18^F]TFAHA volume of distribution data with cocaine group (low-cocaine or high-cocaine) and time (baseline or post-cocaine) as main factors with matching values stacked, full interaction modeling, and Bonferroni’s multiple comparisons post hoc test.

## Results

### Rats display low-cocaine or high-cocaine intake and other addiction-related behaviors

Fig. 1B, C illustrate that rats serendipitously show low-cocaine or high-cocaine intake across 12 days of cocaine-IVSA, which we capitalize on by creating these two groups based on clear differences in behavior. Fig. 1B shows a time course for the 12-day cocaine-IVSA procedure plotting total cocaine infusions. A 2-way RM-ANOVA found no significant interaction for time and group and no significant main effect of time; however, a main effect of group was observed (F_1,6_=85.63, p<0.0001), which indicates the difference between low- and high-cocaine intake is independent of repeated exposure. A *Protected F* post hoc analysis of multiple comparisons in the absence of an interaction or main factor of time was conducted for the total cocaine infusions over time data given the *a priori* hypothesis that cocaine intake would increase over the 12 days of cocaine-IVSA and to follow up on the significant main effect of group. Post hoc analysis revealed that the rats displaying low-cocaine intake were significantly different from those with high-cocaine intake across 3 of the last 4 days of the 12-day cocaine-IVSA procedure (p<0.05). Fig. 1C shows that rats categorized as low-cocaine (<100 total infusions) are significantly different from rats categorized as high-cocaine (>200 total infusions) when comparing the total (summed) cocaine infusions for all 12 days (*t*_6_=9.254, p<0.0001).

**Fig. 2.**
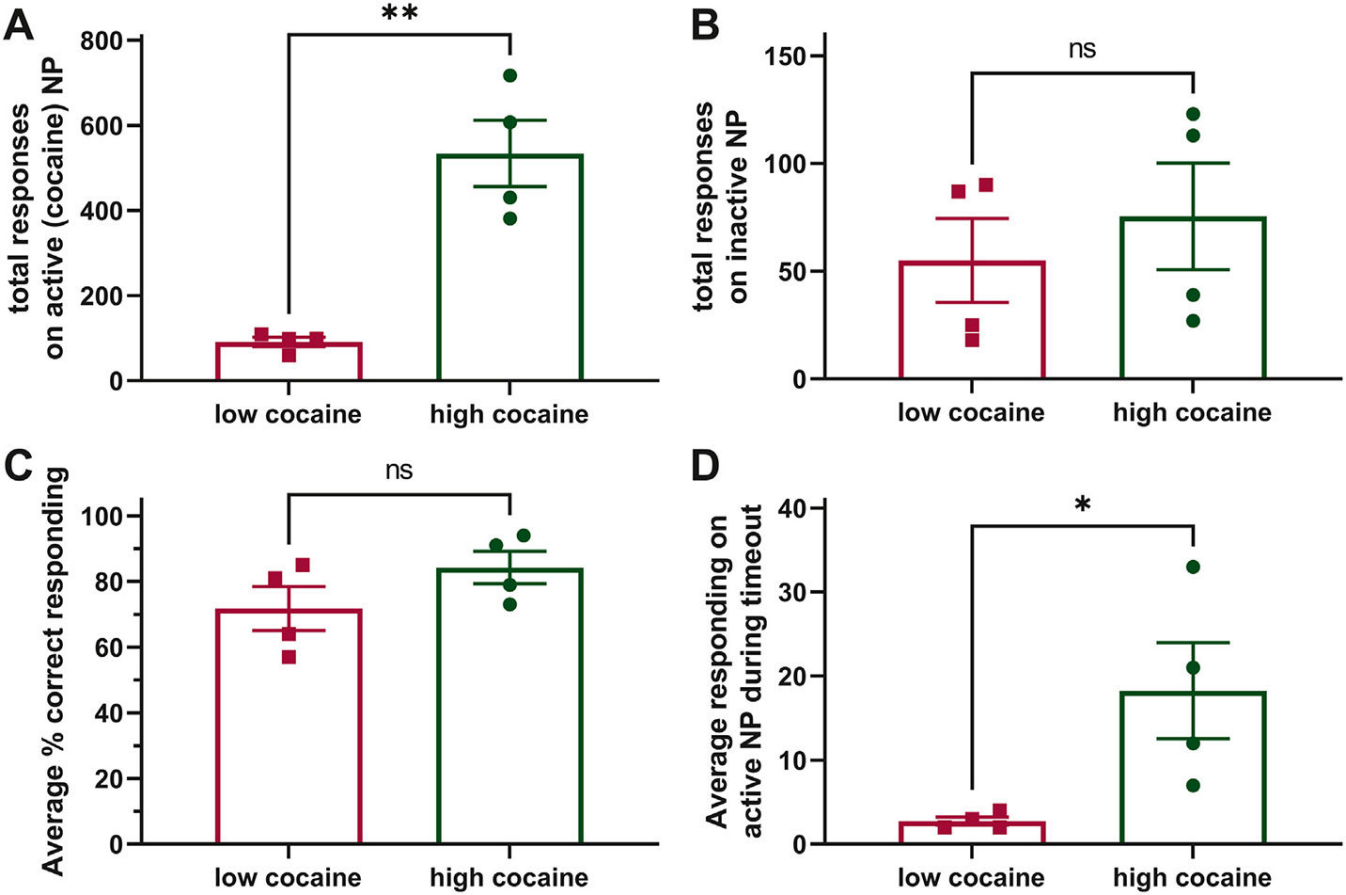
Intravenous self-administration (IVSA) of cocaine by rats taking high cocaine or low cocaine infusions shows compulsive and impulsive behavior in high cocaine taking rats. Male Sprague-Dawley rats were given 3-h access to IVSA of cocaine (0.5 mg/kg/infusion) over 12 days under fixed-ratio 1 (FR1) conditions. (**A)** The total number of responses on the active (cocaine) nose-poke (NP) plotted as the sum of daily activity over the 12 days shows on the active (cocaine) lever was significantly greater in high-cocaine compared to low-cocaine taking rats. These data suggest high-cocaine taking is associated with compulsive behavior. (**B)** The total number of responses on the inactive NP plotted as the sum of daily activity shows no differences between groups. (**C)** The percent correct responding on the active NP compared to the inactive NP plotted as the average daily activity shows high rates of correct responding for both groups with no differences between groups. (**D)** The number of responses on the active NP during the *timeout* period, when the drug is not delivered, plotted as the average daily activity was significantly greater in high-cocaine compared to low-cocaine taking rats. These data suggest high-cocaine taking is associated with impulsive behavior. *p<0.05, **p<0.01, and ns=not significant. The high-cocaine taking group is shown in green color and circle symbols, while the low-cocaine taking group is shown in red and square symbols. All data are presented as group means ± s.e.m. or individual data points (n=4 rats/group).

Fig. 2A-D show supportive data that rats in the low-cocaine and high-cocaine groups are significantly different in other addiction-related behaviors yet similar in general learned behaviors. Fig. 2A shows that compulsive-like behavior, as measured by the total responses on the active (cocaine) NP, was significantly greater in high-cocaine rats compared to low-cocaine rats (*t*_6_=5.615, p=0.0014). Fig. 2B shows that the total responses on the inactive NP are not significantly different between groups despite the difference in active NP responses between groups. Fig. 2C shows that the average percent correct responding was 72% for low-cocaine and 84% for high-cocaine rats with no significant difference between groups. Fig. 2D shows that impulsivity-like behavior, as measured by total active NP during timeouts, was significantly greater in high-cocaine rats compared to low-cocaine rats (*t*_6_=2.707, p=0.0353).

### Spatial, and temporal dynamics of class IIa HDACs expression-activity in the brain of cocaine-IVSA rats

**Fig. 3.**
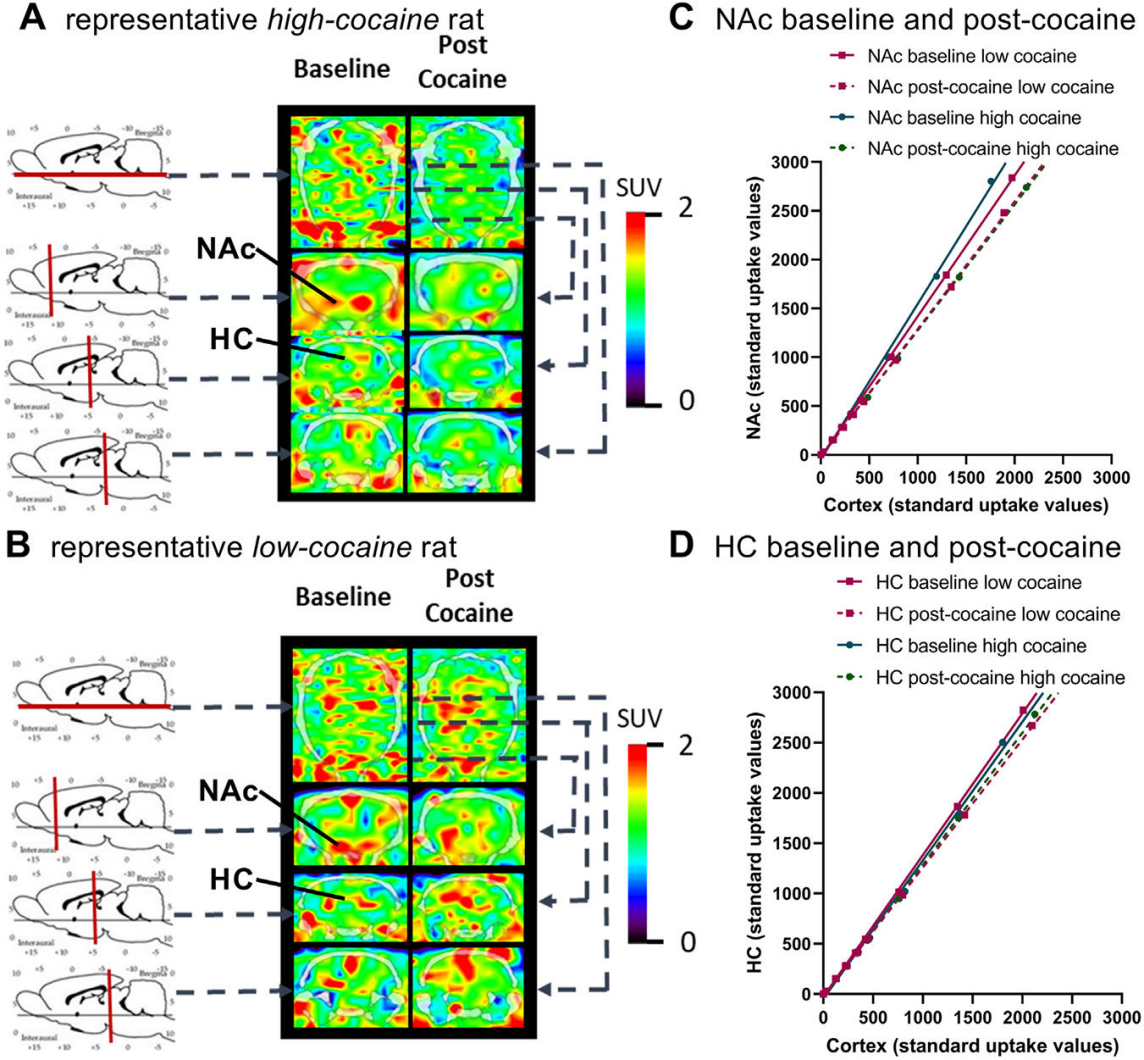
Molecular epigenetic imaging of rats self-administering cocaine using PET/CT with [^18^F]TFAHA. (A and B) Representative [^18^F]TFAHA PET/CT-images of standard uptake values (SUV) in the rat brain from a high-cocaine taking **(A)** and a low-cocaine taking **(B)** rat at 20 min post-radiotracer administration are shown. Locations of axial and coronal sections are indicated by vertical red lines and horizontal black lines on schematics of rat brains, and the relationship between sagittal and coronal PET/CT-imaging is shown by dotted lines on sagittal (top row) and coronal (bottom 3 rows) brain images. (**C and D)** Logan graphical analyses that were used to derive Distribution Volumes for [^18^F]TFAHA for nucleus accumbens (NAc) **(C)** and hippocampus (HC) **(D)**, using cortex as a reference tissue, are shown. Average data points and linear regression analyses for baseline or post-cocaine imaging sessions and those for low-cocaine or high-cocaine taking groups are shown. Calculations for plotting SUV data and deriving [^18^F]TFAHA Distribution Volumes are in Methods.

**Fig. 4.**
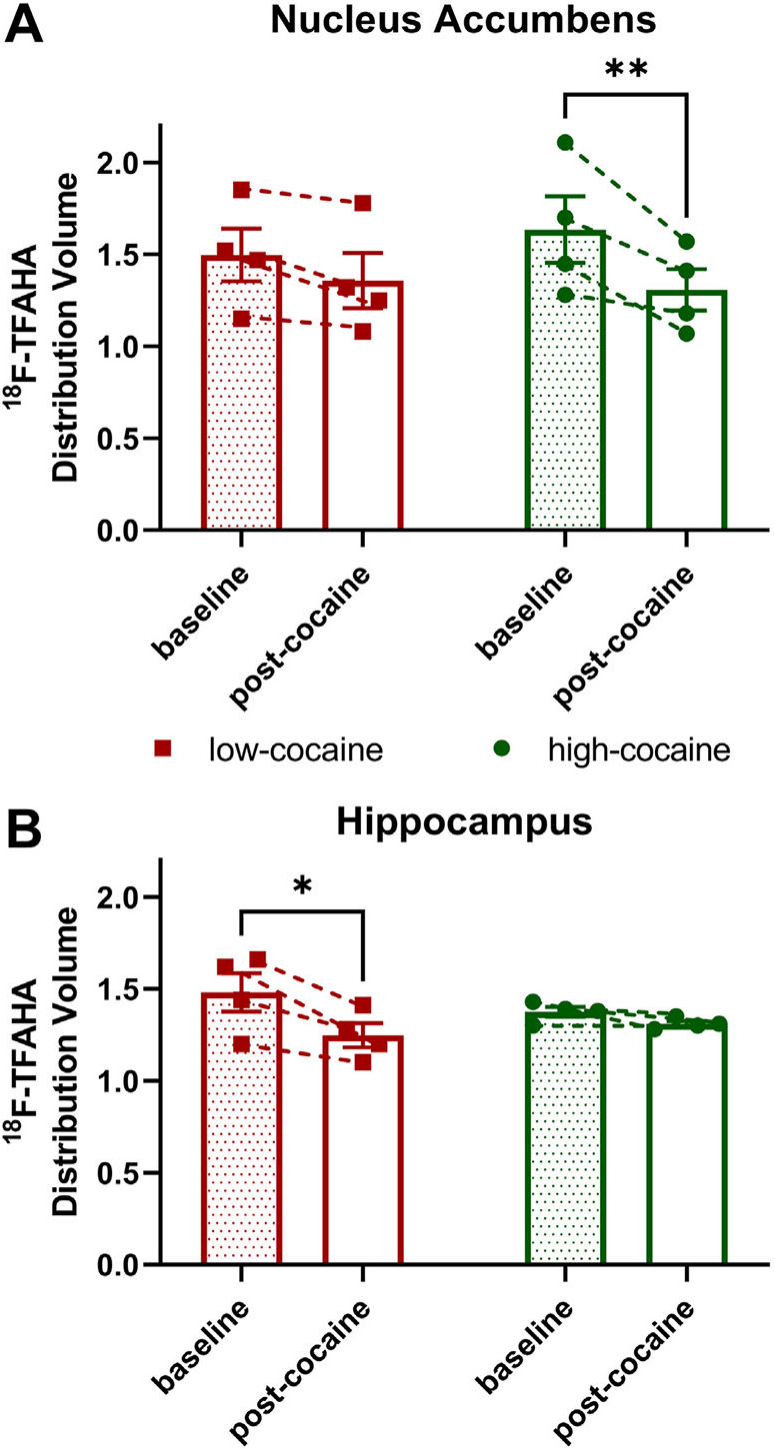
Distribution volumes for [^18^F]TFAHA in rats displaying high-cocaine or low-cocaine intake. (A) In the nucleus accumbens, high-cocaine, but not low-cocaine, taking rats had significantly lower [^18^F]TFAHA accumulation within-subject from baseline to post-cocaine PET-imaging. (**B)** On the other hand, in the hippocampus only low-cocaine taking rats had significantly lower [^18^F]TFAHA accumulation within-subject from baseline to post-cocaine PET-imaging. All data are presented as group means ± s.e.m. or individual data points (n=4 rats/group); *p<0.05, **p<0.01.

Fig. 3A-D display brain images using PET/CT-imaging and Logan graphical analyses (plots) of [^18^F]TFAHA in NAc and HC of rats displaying low-cocaine or high-cocaine intake. Baseline and post-cocaine PET/CT-images of a representative high-cocaine taking rat (Fig. 3A) and a representative low-cocaine taking rat (Fig. 3B) show a sagittal view of NAc and 3 coronal views that include NAc and HC. In addition, Logan plots of NAc (Fig. 3C) and HC (Fig. 3D) data with frontal cortex as reference are shown. These plots were generated using a single compartment model and linear regression analyses of pharmacokinetic data for the reversible uptake of [^18^F]TFAHA radiotracer to yield Distribution Volumes for the [^18^F]-leaving group that represents class IIa HDAC expression-activity shown in Fig. 4.

Fig. 4A, B show [^18^F]TFAHA Distribution Volumes of NAc and HC for groups of rats displaying low-cocaine or high-cocaine intake. A 2-way RM-ANOVA of NAc data found no significant interaction for time and group and no significant main effect of group; however, a main effect of time was observed (F_1,6_=20.51, p=0.0040). Similarly, analysis of HC data found no significant interaction for time and group and no significant main effect of group; however, a main effect of time was observed (F_1,6_=14.92, p=0.0083). *Protected F* post hoc analyses of multiple comparisons in the absence of an interaction or main factor of treatment were conducted for the [^18^F]TFAHA Distribution Volumes from the NAc or HC given the *a priori* hypothesis that cocaine would decrease class IIa HDAC expression-activity in the brain, particularly within-subject over time across the 12-day cocaine-IVSA procedure. Bonferroni’s post hoc analysis of within-subject data (i.e. baseline vs. post-cocaine) from the NAc revealed that the high-cocaine, but not the low-cocaine, group had a significant decrease in [^18^F]TFAHA Distribution Volume following cocaine-IVSA (p=0.0083). On the other hand, post hoc analysis of within-subject data from the HC revealed that the low-cocaine, but not the high-cocaine, group had a significant decrease in [^18^F]TFAHA Distribution Volume following behavioral testing (p=0.0105). Not surprisingly since a main effect of group was not observed in the 2-way RM-ANOVA, post hoc multiple comparisons of between-subject data shows that comparison of low-cocaine and high-cocaine groups at baseline or at post-cocaine found no significant difference in either the NAc or HC.

## Discussion

This study reports the first longitudinal, repeated-measures data obtained *in vivo* to implicate class IIa HDACs in the persistent behavioral effects of cocaine. The well-known dual role of class IIa HDACs as scaffold proteins and as active enzymes provides basis for understanding our observations [26]. Decreases in [^18^F]TFAHA accumulation in NAc of high cocaine taking rats or in HC of low cocaine taking rats may reflect (1) a decrease in levels of class IIa HDACs and/or (2) a decrease in the access to the enzymatic site of class IIa HDACs as they complex with other signaling molecules.

A decrease in levels of class IIa HDACs parsimoniously explains our findings as changes in class IIa HDAC mRNA and protein levels in the brain *ex vivo* have been observed following cocaine and other psychostimulant administration in rats. The actions of amphetamines and cocaine that are mediated by immediately early genes and HDAC class I and IIa enzymes have been recently reviewed by Bisagno and Cadet [27]. Repeated methamphetamine leads to HDAC4- and HDAC5-dependent increased H4-acetylation in prefrontal cortex as determined by ChIP-PCR in mice, likely reflecting decreased HDAC4 or HDAC5 activity [28]. Transgenic mice lacking HDAC4 and/or HDAC5 also implicate the role of class IIa HDACs in the persistent behavioral effects of cocaine likely by affecting associative learning [5,19]. Importantly, Nester and colleagues have shown that loss of HDAC5 in NAc sensitizes neuronal responses to chronic, but not acute, cocaine administration, suggesting that HDAC5 in the reward system is a key regulator in the transition to cocaine dependence [11]. The dynamics of class IIa HDACs during prolonged withdrawal from methamphetamine in rats supports this hypothesis as HDAC4 and HDAC5 mRNA levels are increased in Fos-based neuronal ensembles within the dorsal striatum [4].

A known role of class IIa HDACs exists for the effects of other abused substances, as mediators in the vulnerability to addiction, and in regulating glial response to drugs of abuse. For example, caffeine administration has also been shown to decrease phosphorylated-HDAC4 and phosphorylated-HDAC5 in the prefrontal cortex of mice, decrease HDAC5 in N2a cells, and decrease levels of total and phosphorylated HDAC7, another class IIa HDAC, in the prefrontal cortex of mice [3]. Not surprisingly, decreased levels of HDAC4 and HDAC5 have also been implicated in the gateway-based effects that nicotine and alcohol have on subsequent cocaine use. The mechanism of alcohol or nicotine involves inhibition or proteasome-mediated degradation of nuclear class IIa HDACs in the NAc, which increases global and specifically H3-acetylation leading to epigenetic changes that promotes the behavioral effects of cocaine [29,30]. Interestingly, using human primary astrocytes, exposure to cocaine, methamphetamine, or morphine increased HDAC4 and decreased HDAC5 levels, and a differential drug response was observed for HDAC7 with cocaine increasing and morphine decreasing levels [7]. The common theme among these studies appears to be decreased class IIa HDACs, commonly HDAC4 and HDAC5, levels in the NAc during active drug-taking or administration leading to enhanced histone acetylation in the brain’s reward network and the addiction-related behavioral effects caused by drugs of abuse in rodents.

Alternatively, a decrease in access to the enzymatic site of class II HDACs could also account for our PET-imaging findings using [^18^F]TFAHA, which is a substrate for the deacetylase enzymatic-site of class II HDACs [20,22]. Research by Cowan and colleagues has shown that cocaine causes HDAC5 to translocate to the nucleus, while HDAC4 undergoes nuclear export. In the nucleus, HDAC5 complexes with HDAC3 to indirectly deacetylate histones and promote spinogenesis, whereas in the cytoplasm, HDAC4 plays a role in cocaine-mediated behaviors yet its mechanism remains to be defined [12,14,31]. The enzymatic activity of HDAC5 is influenced by complexing with HDAC3 and other transcriptional regulators, which indirectly limits accessibility to the catalytic site of HDAC5 [21,32,33]. For our PET-imaging studies, this molecular action of HDAC5 would result in less accumulation of the [^18^F]TFAHA product, which is our observation in NAc following high-cocaine taking or in HC after low-cocaine taking.

The effect on class IIa HDAC expression-activity in NAc or HC of rats self-administering cocaine that we observed using PET-imaging may be reflective of the behavior that each brain region is known to regulate. The aforementioned studies illustrate the role of HDAC4 and HDAC5 in the NAc and in the prefrontal cortex on the locomotor activating, rewarding, reinforcing, and motivational effects of cocaine. Our observations are congruent with these publications as those rats with high-cocaine taking displayed significantly decreased class IIa HDAC expression-activity in the NAc, but not in the HC. On the other hand, the rats with low-cocaine self-administration had a significant decrease in class IIa HDAC expression-activity in HC, but not in NAc. The low expression-activity of class IIa HDACs in the HC may disrupt drug-context associations thereby minimizing cocaine-taking behaviors. Indeed, we have previously shown that male rats exposed to single prolonged stress, a model of posttraumatic stress disorder (PTSD), and displaying deficient fear-extinction memory recall had decreased HDAC4 and HDAC5 levels in the HC, but not the striatum [34]. In support, Maximov and colleagues found that double knockout of HDAC4 and HDAC5 limits conditioned fear behavior, as well as Barnes maze spatial learning and cocaine conditioned place preference, and that genetic deletion of these epigenetic regulators disrupts morphological and physiological characteristics of excitatory neurons in the HC [19]. A better understanding of the role of class IIa HDACs in distinct brain regions, neural systems, neuro-circuits, or neuronal ensembles that mediated these complex behaviors remains to be elucidated.

Despite our best efforts to minimize limitations to this study, a few aspects of the study should be considered. For example, limitations include low sample sizes (N) of our experimental groups and the use of only males in this study. Statistically significant results were obtained in cocaine-taking behavior and PET-imaging of [^18^F]TFAHA findings despite lower than anticipated power and small group sizes. While striving for reproducible and rigorous results is essential, the use of animal life and unnecessary need to increase sample sizes when *a priori* hypotheses were addressed led us to limit the number of animals used for this study. Additional studies are warranted to replicate and extend these findings using PET-imaging and other research tools to better understand the mechanistic actions and cellular localization (i.e., nucleus or cytoplasm) of class IIa HDACs in key brain regions, including the NAc and HC that govern drug-taking behaviors. Importantly, future studies need to include both female and male individuals. Clear evidence in humans and rodents indicates sex as an essential biological variable in the pharmacodynamics of cocaine [35] and other drugs of abuse [36] as reviewed by Becker and colleagues [37-41]. Also, estrous cycle influences HDAC4 levels in women with PTSD and female mice undergoing fear conditioning, which underscores the influence of sex hormones on brain and behavior relationships [42].

In summary, these longitudinal results obtained *in vivo* are consistent with previous *ex vivo* reports demonstrating that the effects of cocaine taking and seeking are associated with altered expression-activity of HDACs class IIa enzymes in the NAc and HC. PET/CT-imaging with [^18^F]TFAHA has proven effective for non-invasive, quantitative, and repetitive monitoring of HDACs class IIa expression-activity in a cocaine self-administration model in rats and may facilitate the development and clinical translation of epigenetics–targeted therapies of cocaine addiction. These findings support the ability of PET-imaging with [^18^F]TFAHA as an *in vivo*, translational research tool to measure class IIa HDACs and potentially aid in treatment monitoring and unique clinical approaches for cocaine use disorder. For example, longitudinal studies using [^18^F]TFAHA with PET-imaging could be conducted to build a database of class IIa HDAC expression-activity patterns across the whole brain that could be used as a tool to predict the propensity of an individual to use drugs in high amounts. Furthermore, [^18^F]TFAHA with PET-imaging could be combined with other imaging methods done *in vivo*, such as functional magnetic resonance imaging or photoacoustic imaging, to determine the role of class IIa HDACs in relationship to neural activity in response to cocaine or other substances in animals or humans.

## Data Availability

Data will be made available on request.
